# Molecular detection of *Leptospira* spp. in small wild rodents from rural areas of São Paulo State, Brazil

**DOI:** 10.1590/0037-8682-0160-2023

**Published:** 2023-09-22

**Authors:** Evelyn Cristine da Silva, Felipe Fornazari, João Marcelo Azevedo de Paula Antunes, Larissa de Castro Demoner, Lucia Helena O’Dwyer de Oliveira, Marina Gea Peres, Jane Megid, Helio Langoni

**Affiliations:** 1 Universidade Estadual Paulista, Instituto de Biotecnologia, Botucatu, SP, Brasil.; 2 Universidade Estadual Paulista, Departamento de Produção animal e Medicina Veterinária Preventiva, Botucatu, SP, Brasil.; 3 Universidade Federal Rural do Semiárido, Departamento de Ciência Animal, Mossoró, RN, Brasil.; 4 Universidade Estadual Paulista, Departamento de Biodiversidade e Bioestatística, Instituto de Biociências, Botucatu, SP, Brasil.; 5 Centro Universitário Sudoeste Paulista, Avaré, SP, Brasil.

**Keywords:** leptospirosis, qPCR, Wildlife, Renal carrier

## Abstract

**Background:**

Leptospirosis represents a One Health issue, affecting humans and animals. This study investigated pathogenic leptospires in small wild rodents in São Paulo, Brazil.

**Methods:**

Kidney samples from 164 rodents underwent qPCR testing, targeting pathogenic *Leptospira* spp.

**Results:**

Thirty-five animals (21.34%) tested positive, including five rodent species: *Akodon montensis* (2/21; 9.5%), *Necromys lasiurus* (1/4; 25%), *Oligoryzomys nigripes* (24/92; 26.1%), *Oligoryzomys flavescens* (5/26; 19.2%), and *Sooretamys angouya* (3/14; 21.4%). Botucatu municipality exhibited the highest prevalence, with 42.5% (20/47) of the animals testing positive.

**Conclusions:**

The presence of *Leptospira* spp. in wild rodents suggests they may be chronic carriers, contaminating the environment.

Leptospirosis is a zoonotic disease of significant consequence, affecting both animals and humans^1^. Rodents, particularly the species *Rattus norvegicus*, commonly known as the rat or sewer rat^1^, are the primary reservoirs and transmitters of leptospirosis to humans1. This species is the natural host of *Leptospira interrogans* serovar Icterohaemorrhagiae, which is considered the most significant agent for public health^2^. Other urban rodents, such as *Mus musculus* and *Rattus rattus*, are also identified as reservoirs of various leptospire strains^3^. This observation extends to the hundreds of wild rodent species worldwide, with a vast diversity of leptospires reported in these rodents across diverse ecosystems^4^. Human leptospirosis cases have been linked to wild rodents, as evidenced in Southeast Asia^5^. In Thailand, the rodent *Bandicota indica* was identified as the maintenance host of a new variant of *L. interrogans* serovar Autumnalis, which has been responsible for the emergence of leptospirosis outbreaks in humans^6^. Despite their scarcity, studies on leptospirosis in wild rodents have unveiled crucial epidemiological characteristics of this disease, impacting not only public health but also the transmission mechanisms of leptospires^3^ ^,^ ^4^.

Several studies have examined leptospirosis in wild rodents in Brazil. One of the most significant studies identified rodents from the *Akodon* and *Oligoryzomys* genera as carriers of leptospires in the Atlantic Forest biome^7^. Another study in the Western Amazon in Brazil reported a high prevalence of *Leptospira* spp. detection in small mammals from the Didelphimorphia and Rodentia orders^4^. Consequently, research on leptospires in wild rodents is crucial for understanding the epidemiological aspects of this bacterium, including infection prevalence, the most common serogroups of leptospires, and the primary animal reservoirs that can serve as infection sources. A comprehensive understanding of this zoonosis necessitates analyses that consider the interplay between humans, animals, and the environment, aligning with the One Health^4^ concept^4^ ^,^ ^7^. These aspects have been extensively explored in other countries, leading to the discovery of new epidemiological characteristics of this significant zoonosis. Therefore, comprehending the factors associated with leptospirosis in various Brazilian biomes remains a considerable challenge, and studies focusing on this topic are vital. This study aimed to detect pathogenic leptospires molecularly in kidney samples from small wild rodents in four municipalities in the state of São Paulo, Brazil. 

The study examined wild rodents from the municipalities of Torre de Pedra (23°14'58”S 48°11'39”W), Bofete (23°05'54”S 48°11'26”W), Anhembi (23°05'54”S 48°11'26”W), and Botucatu (22°53'25”S 48°27'19”W). These municipalities are situated in the central region of São Paulo, southeastern Brazil. This unique geographic area is characterized by the transition between the Cerrado and Atlantic Forest biomes, with the semideciduous seasonal Atlantic Forest being the predominant vegetation. The rodents were captured in various forest fragments on different rural properties. The capture period spanned from September 2011 to June 2014. The study analyzed 164 small wild rodents of various species, as detailed in Table 1.

**TABLE 1:** Species, origin, and results of wild rodents submitted to molecular diagnosis of *Leptospira* spp.

Species of rodent	Total analyzed	Positive animals	%	95% CI	City Positive animals/total analyzed/% (95% CI)			
					Botucatu	Anhembi	Bofete	Torre de Pedra
*Akodon montensis*	21	2	9.5	1.2-30.4	2/14/14.3 (1.8-42.8)	0/0/0	0/7/0 (0.0-41.0)	0/0/0
*Oligoryzomys nigripes*	92	24	26.1	17.5-36.3	16/27/59.2 (38.8-77.6)	7/46/15.2 (6.3-28.8)	1/15/6.6 (0.1-31.9)	0/4/0.0 (0.0-60.2)
*Oligoryzomys flavescens*	26	5	19.2	6.5-39.3	1/2/50.0 (1.2-98.7)	2/14/14.3 (1.8-42.8)	2/10/20.0 (2.5-55.6)	0/0/0
*Necromys lasiurus*	4	1	25.0	0.6-80.6	1/3/33.3 (8.4-90.5)	0/0/0	0/1/0 (0.0-97.5)	0/0/0
*Nectomys squamipes*	3	0	0.0	0.0-70.7	0/3/0 (0.0-70.7)	0/0/0	0/1/0 (0.0-97.5)	0/0/0
*Sooretamys angouya*	14	3	21.4	4.6-50.8	0/1/0 (0.0-97.5)	2/12/16.6 (2.1-48.4)	1/1/100.0 (2.5-100)*	0/0/0
*Calomys tener*	4	0	0.0	0.0-60.2	0/0/0	0/2/0 (0.0-84.2)	0/2/0 (0.0-84.2)	0/0/0
**Total**	**164**	**35**	21.3	**15.3-28.4**	**20/47/42.5** (28.2-57.8)	**11/76/14.4** (7.4-24.4)	**4/37/10.8** (3.0-25.4)	**0/4/0.0** (0.0-60.2)

**%:** percentage of positive animals in relation to the total analyzed. **95% CI:** 95% confidence interval by binomial distribution. **97.5%:** one-sided confidence interval.

The study received approval from the Ethics Committee on Animal Use at the Faculty of Veterinary Medicine and Zootechnics/UNESP-Botucatu (CEUA Protocol No. 112/2010), the Chico Mendes Institute for Biodiversity Conservation (SISBIO Protocol Nos. 36283-3 and 23918-1), and the Ethics Committee for the Use of Animals at the Faculty of Veterinary Medicine and Zootechnics/UNESP-Botucatu (CEUA Protocol No. 0072/20).

Genomic DNA was initially extracted from the samples for molecular analysis using the Illustra TissueMini SpinKit® (GE Healthcare), following the manufacturer’s instructions. The quality of DNA extraction and the presence of molecular diagnostic inhibitors were verified by performing conventional Polymerase Chain Reaction (PCR) to amplify the glyceraldehyde-3-phosphate dehydrogenase (GAPDH) gene in all study samples. This was done using the GAPDH-F 5´-CCTTCATTGACCTCAACTACAT-3´ and GAPDH-R 5´-4CCAAAGTTGTCATGGATGACC-3’ primers as previously described^8^. The molecular detection of *Leptospira* spp. was carried out using a qPCR that targeted the lipL32 gene, which is associated with leptospirosis pathogenesis. This gene, which shows high conservation among *Leptospira* serovars with similarity percentages ranging from 93% to 99%^1^, was amplified using the lipL32-F (5'-AAGCATTACCCGCTTG TGGTG-3') and lipL32-R (5′-GAACTCCCATTTCAGCGATT-3′)^9^ primers. The qPCR reaction involved the use of 5 μL Power SYBR®Green PCR Master Mix (Applied Biosystems®), 3.8 μL nuclease-free water, and 0.1 μL (10 μM) of each primer. The extracted sample volume added to the reaction was 1 μL, making the total volume per reaction 10 μL. All samples were tested in duplicate. The reaction thermocycling conditions were set at 95 °C for 10 minutes, followed by 45 cycles at 95 °C for 10 seconds and at 58 °C for 30 seconds, and a final melting curve step of 55 minutes (60 °C to 95 °C). The qPCR was conducted using the StepOnePlus™ Real-Time PCR System (Applied Biosystems®), and the StepOne v2.1 software was used to read the DNA amplification and dissociation fluorescence. Samples were deemed positive when the dissociation temperature varied by a maximum of 1 °C compared to the positive control, as depicted in Figure 1.

**FIGURE 1:**
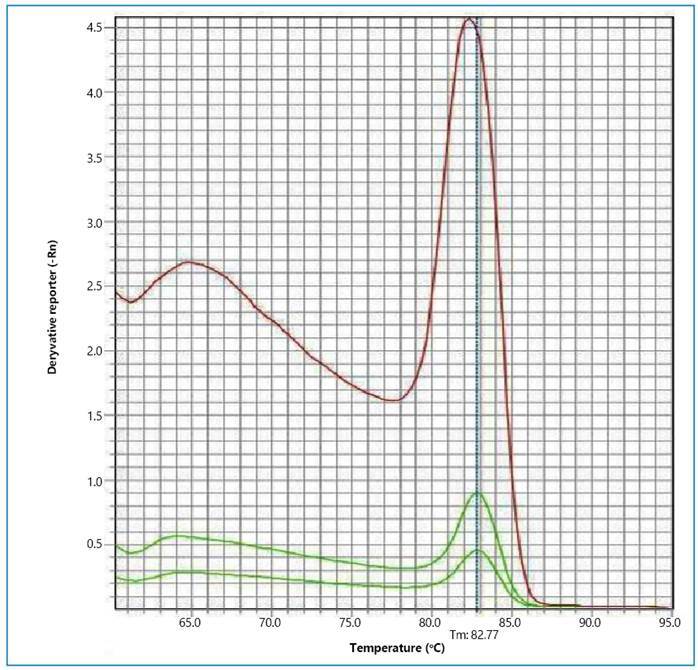
Result of molecular detection of *Leptospira* spp. Dissociation fluorescence of DNA amplified by real-time polymerase chain reaction (qPCR). Red curve: positive control whose dissociation temperature is 82.77 °C; green curves: wild rodent kidney sample with DNA amplification showing a dissociation temperature compatible with the positive control.

The PCR of the GAPDH gene yielded positive results for all samples. Out of the studied rodents, 35 (21.3%) tested positive, with the species *Oligoryzomys nigripes* exhibiting the highest prevalence at 26.1%. Positive cases were recorded in all municipalities except for Torre de Pedra, with Botucatu registering the highest prevalence at 42.5% (20/47). These results are consolidated in Table 1.

In this study, we discovered a significant proportion of small wild rodents harboring leptospires, suggesting their potential role as infection sources within their respective biomes. Other research involving wild rodents has reported an average prevalence of 7.1% in Southeast Asia^5^, 5.9% in Germany^10^, and 7.7% in the Seychelles, Africa^11^. A study in the state of Rio de Janeiro found that 28% of the analyzed wild rodents tested positive for *Leptospira* spp. via PCR^7^. A recent investigation in the Amazon reported a high prevalence of 44.7% in PCR kidney samples from carriers/hosts of 16 species within the Didelphimorphia and Rodentia^4^. The findings of this study align with those in existing literature, highlighting the widespread distribution of leptospires among various species of small wild rodents across different countries and biomes. The prevalence identified in this study (21.3%), coupled with the results of two recent Brazilian studies, suggests that the leptospiral infection rate in wild rodents in Brazil exceeds that in other countries.

The majority of the 24/35 positive animals (68.57%) belonged to the *O. nigripes* species. This species, along with the *Akodon* and *Oligoryzomys* genera, has been identified as a carrier of pathogenic leptospires^6^ ^,^ ^12^. *O. nigripes* is one of the most prevalent and adaptable wild rodents in Brazil, potentially enhancing its role as a source of leptospiral infection. These traits are also found in urban rodents, which are deemed significant reservoirs of leptospirosis for humans. 

The animals examined in this study were sourced from transitional biomes between the Atlantic Forest and the *Cerrado*. The high percentage of positive rodents could be attributed to environmental conditions that promote the survival of leptospires outside their hosts, such as elevated temperatures and soil moisture^12^. In other biomes, pathogenic leptospires have been identified in wild rodents, for instance, in the Mediterranean forests of Chile^12^ and the Argentine pampas^13^. The *Cerrado* biome has been subject to limited studies concerning diseases prevalent in its fauna, despite its global ecological significance due to its highly biodiverse tropical savannas^14^. Certain species of wild animals, excluding small rodents, have also been reported as leptospires reservoirs in the Botucatu municipality^15^, thereby affirming the widespread distribution of this bacterium in the region’s fauna. It is noteworthy that the 16% prevalence reported in a separate study in the same region^15^ is comparable to the 21.3% found in this study. The variation in results between municipalities could be linked to diverse geographic factors in each region, underscoring the necessity for more extensive studies to delineate the epidemiology of *Leptospira* spp. among wild rodents. Further research is also needed to clarify the role of wildlife in the epidemiology of leptospirosis across different Brazilian biomes, encompassing public, animal, and environmental health. Future studies should prioritize genetic analyses of leptospires from various sources and explore epidemiological variables associated with diagnostic outcomes. 

This study employed the qPCR technique to detect genetic material from pathogenic leptospires. Despite its high cost, this technique is widely used due to its simplicity and effectiveness. Molecular methods are often recommended for investigating the epidemiology of leptospirosis in wild animals^7^ ^,^ ^15^, as they facilitate the identification of the renal carrier state. Conversely, serological techniques demonstrate low seropositivity in small wild mammals, rendering them unsuitable for research on wild species. Infected animals may even present as seronegative^7^. Consequently, this study refrained from using serological techniques for two reasons: firstly, as previously mentioned, they are inefficient in identifying *Leptospira* carriers, and secondly, the limited availability of serum due to the small size of the animals. Blood collection from these animals is often challenging and, even when successful, yields a minimal volume. 

Leptospirosis in wild animals plays a significant role in One Health approaches. Wild species can contract the disease and/or serve as infection sources for humans and domestic animals. Environmental characteristics are crucial in determining leptospires’ “survival outside their hosts, which influences infection rates among humans and animals exposed to contaminated water and soil^7^ ^,^ ^15^. Therefore, comprehensive, transdisciplinary, and integrated interventions are essential for investigating leptospirosis and directing preventive measures. 

In conclusion, various species of small wild rodents were identified as carriers of *Leptospira* spp. In the central region of São Paulo state. A notable prevalence was observed, particularly among the *O. nigripes* species. Among the four municipalities studied, Botucatu exhibited the highest prevalence.
